# Climate and Gender: Association Between Droughts and Intimate Partner Violence in India

**DOI:** 10.1093/aje/kwad222

**Published:** 2023-11-15

**Authors:** Nabamallika Dehingia, Lotus McDougal, Jay G Silverman, Elizabeth Reed, Lianne Urada, Julian McAuley, Abhishek Singh, Anita Raj

**Keywords:** drought, India, intimate partner violence, violence against women

## Abstract

Extreme climate events are related to women’s exposure to different forms of violence. We examined the relationship between droughts and physical, sexual, and emotional intimate partner violence (IPV) in India by using 2 different definitions of drought: precipitation-based drought and socioeconomic drought. We analyzed data from 2 rounds of a nationally representative survey, the National Family Health Survey, where married women were asked about their experiences of IPV in the previous year (2015–2016 and 2019–2021; *n* = 122,696). Precipitation-based drought was estimated using remote sensing data and geographic information system (GIS) mapping, while socioeconomic drought status was collected from government records. Logistic regression models showed precipitation-based drought to increase the risk of experiencing physical IPV and emotional IPV. Similar findings were observed for socioeconomic drought; women residing in areas classified as drought-impacted by the government were more likely to report physical IPV, sexual IPV, and emotional IPV. These findings support the growing body of evidence regarding the relationship between climate change and women’s vulnerability, and highlight the need for gender responsive strategies for disaster management and preparedness.

## Abbreviations

aOR: adjusted odds ratio
CHIRPS: Climate Hazards Group InfraRed Precipitation with Station
CI: confidence interval
GIS: geographic information system
IPV: intimate partner violence
NFHS: National Family Health Survey
PSU: primary sampling unit

##  

Extreme weather events, including droughts, have adverse effects on people’s well-being, including physical health, mental health, water and sanitation, as well as the experience of interpersonal violence (1). Women however, are at a higher risk of experiencing the burden of such natural hazards, owing to multiple social, political, and cultural factors (2, 3). For example, in many low- and middle- income countries, women carry the primary responsibility of gathering firewood, fetching water, and other tasks that require interactions with natural resources affected by drought. Women in rural areas are at particular risk, as there is typically more interface with the environmental risks. There is also a growing body of research highlighting the role of extreme climate shocks in women’s experience of violence, including intimate partner violence (IPV), sexual assault, female genital mutilation, honor killing, and the trafficking of women (4–7). Researchers attribute these to the increases in deprivation and conflict due to climate shocks. In this study, we extend this literature and test for an association between droughts and IPV among married women in India (8).

Few studies have examined the relationship between droughts and IPV, and those that have offer findings that are mixed and inconclusive. An analysis of data from 19 countries in Africa found a significant and positive relationship between droughts and physical IPV (9), but Cooper et al.’s (10) study with 63 countries across Latin America, Asia, and Africa found no evidence in support of the hypothesized relationship. While large-scale, multicountry studies can provide valuable insights on global trends, they can often mask the variations within results across smaller regions. This could be due to the regional and cultural variations in coping strategies for droughts, such as the intensity or timeframe of drought, related consequences within communities, and the extent of drought induced migration (11). Differences might also exist across regions in terms of the impacts of climate shocks on gender norms and gender roles within families. It is thus imperative to also study droughts and IPV, at subregional or national scales, for a local understanding of this issue.

### Drought has multiple definitions

One key challenge when studying the gendered impacts of droughts is the selection of an appropriate definition of drought. Droughts are complex events with varying characteristics manifesting across different agroclimatic regions. Drought is defined as a period of unusual dry weather that causes hydrological imbalances and/or crop damage. Broadly, there are 4 different ways that droughts can be defined. Precipitation-based or meteorological droughts measure the departure of the amount of precipitation from average or normal levels. Hydrological droughts refer to dry conditions that lead to a reduction in surface and subsurface water levels. Agricultural droughts capture reduction in moisture levels in the soil that do not meet the requirements of crops, and socioeconomic droughts refer to changes in supply and demand of economic goods, including agricultural production, food grains, crops, forage, etc., as a result of reduced rainfalls (12). To our knowledge, all prior quantitative assessments of the relationship between drought and IPV have used meteorological drought, which estimates the relative level of precipitation as the only definition for droughts (9, 10, 13). This indicator fails to capture any potential variation in actual impacts felt by community members due to the rainfall-related aberrations. Our study uses 2 separate definitions of drought—meteorological or precipitation-based drought and socioeconomic drought—for a robust assessment of the relationship between drought and IPV.

### Drought and IPV in India

The present study focuses on India—an important region for studying droughts and IPV, both of which are highly prevalent in the country. One in every 3 women in India has experienced some form of IPV at least once in her lifetime (14). Droughts are also commonplace; the country experienced 2 major drought periods since 1990, in 1997–2004 and 2011–2015 (15). According to a United Nations global report, India is one of the severely drought-impacted countries for 2020–2022, with two-thirds of the country’s land area experiencing droughts. In addition to extreme health and social impacts, drought episodes during 1998–2017 were estimated to reduce India’s gross domestic product by 2%–5% (16). There are also significant differences across the country with regard to precipitation levels and drought status, and in many areas not historically prone to drought, precipitation levels have been decreasing consistently over the past 10 years (17).

However, limited quantitative research has assessed the risk of climate shocks on violence against women in India, with a majority studies focusing on natural disasters such as the 2004 Indian tsunami (18–20). One study found reduction in rainfall levels to be significantly associated with increased levels of dowry deaths in India (i.e., suicide or murder following marriage related to dowry dissatisfaction) (21). Only one quantitative study has examined the relationship between drought and IPV in India, although this research was limited to 10 historically drought-prone states in the country, and does not provide nationally representative results. The study also relies on a single definition of drought. Findings indicate drought exposure to be associated with an increased risk of physical IPV, and no associations for sexual and emotional IPV (22).

We extend and build on the current literature from India on droughts by using nationally representative, repeated cross-sectional data sets of women and 2 distinct definitions of drought. We hypothesized that women residing in drought-affected areas are more likely to report victimization from physical, sexual, and emotional IPV in the previous 1 year. Findings from this work can support IPV prevention programming and improve our understanding of the intersections of environmental change and violence against women.

## METHODS

### Data

We used data from 2 separate rounds of the cross-sectional survey, Demographic Health Survey (DHS) of India, called the National Family Health Survey (NFHS); it is a nationally representative household survey conducted in 2015–2016 (NFHS-4) and 2019–2021 (NFHS-5). The surveys interviewed women aged 15–49 years on a number of aspects including their sociodemographic characteristics and health behaviors; the survey design has been described elsewhere (14). The surveys used a stratified 2-stage cluster sampling design, selecting first a sample of primary sampling units (PSU) using probability proportional to size, followed by the selection of a sample of households within each PSU using systematic sampling. NFHS provides the geolocation for each surveyed PSU. A subsample of the selected women were asked questions related to agency, mobility, household dynamics, and their experience of spousal violence. Our analytical sample was limited to this subsample of women.

We relied on 2 data sources to capture drought status: 1) satellite imagery data for precipitation-based drought, and 2) government records for socioeconomic drought. Precipitation-based drought status was calculated at the PSU level using GIS mapping. These estimates were based on data from Climate Hazards Group InfraRed Precipitation with Station (CHIRPS) data, which combines satellite imagery with weather station data to create daily precipitation estimates in millimeters at 0.05-decimal-degree resolution. We extracted CHIRPS daily data through the Google Earth Engine platform. Of the 18,798 PSUs, 73 had missing precipitation data in the CHIRPS database and were excluded from our analysis. Publicly available annual government records that classified districts in India as drought-affected were used for creation of the variable related to socioeconomic drought.

To examine the relationship between precipitation-based drought and IPV, the final sample included married women cohabiting with their husbands who responded to the relevant questions in NFHS-4 and NFHS-5 (*n* = 122,696). For testing the relationship between socioeconomic drought and IPV, we used data from eligible women in NFHS-4 only (*n* = 62,464), due to unavailability of district level drought-related information beyond 2016.

### Measures

#### Outcome assessment.

The study included 3 key outcome variables: physical IPV, sexual IPV, and emotional IPV in the 12 months preceding the survey, captured using standard and validated measures of the Demographic Health Surveys. Women were asked if, in the previous 12 months, they had experienced different forms of marital violence “often,” “sometimes,” or “not at all.” Physical IPV covered the respondent (woman) being pushed, being shaken, having something thrown at her, being slapped, being punched or hit with something harmful, being kicked or dragged, being strangled or burnt, being threatened by a knife or gun, or having her arm twisted or hair pulled. Sexual IPV included being forced into unwanted sex or sex acts. Emotional violence included being humiliated, threatened with physical harm, or insulted.

#### Exposure assessment.

**Figure 1:**
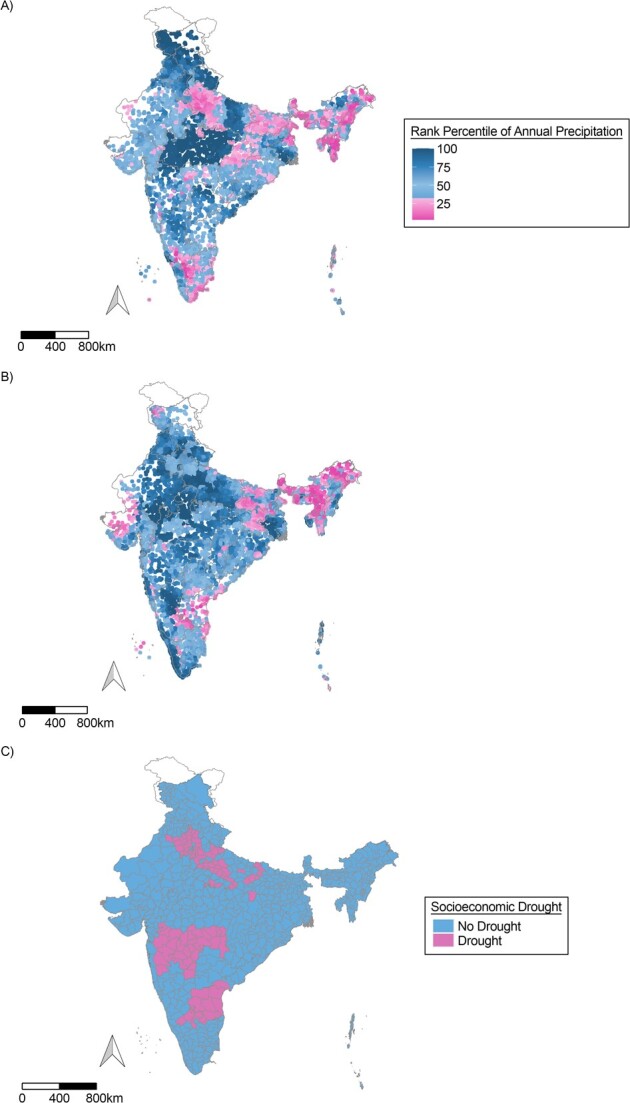
Prevalence of precipitation-based and socioeconomic drought in India for 2013–2014 and 2017–2019. A) Rank percentile of annual precipitation in 2013–2014; B) rank percentile of annual precipitation in 2017–2019; C) socioeconomic drought in 2013–2014. Data source: Climate Hazards Group InfraRed Precipitation with Station data (CHIRPS) extracted from Google Earth Engine. Each dot in (A) and (B) represents a primary sampling unit (PSU) of the demographic health survey. PSUs with an annual precipitation rank less than 30th percentile are classified as drought areas. Data source for socioeconomic drought is government records.

For the variable on precipitation-based drought, we collected the annual precipitation data from 1983 to 2021 for each PSU of NFHS-4 and NFHS-5, using CHIRPS, using GIS mapping (Figure 1). We followed the operationalization of precipitation-based drought variable provided by Epstein et. al (9). To allow for a lag between the outcome and exposure variable, we first calculated precipitation levels for the 12 months preceding the time frame of the IPV experience (outcome variable). For example, for a woman interviewed in April 2015, precipitation-based drought was estimated based on annual precipitation during April 2013 to March 2014, since the timeframe for IPV experience was April 2014 to March 2015. The annual precipitation was ranked with the prior 31 years’ annual values. Next, based on the percentile of these rankings, observations with precipitation level higher than or equal to the 30th percentile were categorized as “no drought,” and those with precipitation level less than the 30th percentile were classified as “drought regions.”

Socioeconomic drought was captured by a binary variable (drought vs. no drought), based on annual data from government records (23). While there are differences in the way that droughts are identified and declared at the state level in India, overall, the state departments rely on information related to the value of crop production, agricultural supply, and demand to determine drought status (24). Drought status is provided at the district level in India, which is an administrative region that encompasses villages and cities (a single district will cover multiple PSUs of the NFHS). Since government records provide drought status yearly, in our sample, for women who were interviewed in 2015, drought status was identified for 2013, and for those interviewed in 2016, drought status was identified for 2014. As noted prior, the analysis with socioeconomic drought was limited to data from NFHS-4, due to unavailability of district-level drought information beyond 2016.

#### Covariates.

Covariates were identified as the minimal sufficient adjustment set from directed acyclic graphs (25) (Web Material, available at https://doi.org/10.1093/aje/kwad222), which considered all possible paths between drought and IPV. The following variables were included as covariates in our models: wealth status, caste (a marker of social class in India), religion (categorized as Hindu, Muslim, and others), woman’s/respondents’ schooling (categorized as <5 years, 5–12 years, >12 years), child marriage (married before 18 years of age vs. married at or after 18 years of age), husband’s occupation (no work, agriculture, others), type of residence (categorized as rural and urban), and a binary variable for whether an area is drought-prone, (i.e., experienced droughts frequently, historically). The models also adjusted for district-level fixed effects, to adjust for any time-invariant unobserved confounders. A composite measure of a household’s cumulative living standard (wealth index) was provided by the NFHS. It was calculated using principal component analysis on variables capturing household’s ownership of selected assets. This continuous index of relative wealth was divided into 5 wealth quintiles, which constituted the wealth status variable. The variable on caste has been categorized as scheduled caste/scheduled tribe, other backward class (a Government of India classification category), and other caste. The variable on drought-prone status is based on data from the government of India’s Drought Prone Areas Programme (DPAP 2010), which classifies regions as drought-prone based on consistent drought events (26). Our sample includes 185 districts classified as drought-prone. These areas receive government assistance to address expected drought consequences, which would likely have an impact on the way individuals living in this area experience the effects of droughts. These regions differ from the precipitation-based and socioeconomic drought areas, with minor overlaps.

The models with precipitation-based drought as the independent variable, which used 2 rounds of NFHS data, adjusted for a dichotomous variable specifying the round of data collection.

### Analysis

Descriptive statistics have been provided. χ^2^ tests identified binary associations between each exposure variable and the 3 forms of IPV: physical, sexual, and emotional.

The unit of our analysis is the woman. To examine the relationship between precipitation-based drought and IPV, we fitted fixed-effects logistic regression models specified at the district level, to adjust for any time-invariant unobserved confounders. For the analysis with socioeconomic drought as the key exposure, which relied on data from one round of NFHS (NFHS-4), we fitted logistic regression models. The models adjusted for all selected covariates. Prior studies have indicated heterogeneity of effects in the relationship between droughts and IPV according to the socioeconomic status of women (9). We thus fitted limited-sample stratified models to examine any differences in the hypothesized relationships across selected indicators of relevance—wealth status, years of education, husband’s occupation, rural/urban residence, and drought-pronenesss. All models were tested for multicollinearity, and none was observed.

We performed sensitivity checks for the analysis with precipitation-based drought by testing additional models with independent variables: 1) 2-year lag between drought and IPV, and 2) no lag. The results for the variable with 2-year lag did not differ meaningfully from our original model (1-year lag), although the 2-year-lag model showed poorer fit relative to the original model. The model with no lag between drought and IPV showed no significant associations, indicating that the impacts of drought with regard to gender-based violence perhaps take time to manifest and observe (results not shown). This is expected, given our understanding from prior literature that droughts are potentially linked to IPV through reduced income and financial security and increased stress and anxiety. We present the results from these additional models in the Web Material. We used the Google (Mountain View, California) Earth Engine platform to extract CHIRPS data, and R, version 4.1.2 (R Foundation for Statistical Computing, Vienna, Austria), and Stata, version 15.0 (StataCorp LLC, College Station, Texas), for all analyses.

## RESULTS

Approximately 22%, 5%, and 11% of the sampled women in the 2 rounds of NFHS reported to have experienced physical IPV, sexual IPV, and emotional IPV, respectively, in the previous 12 months. Twenty-two percent were living in areas that experienced precipitation level drought in the index year. Thirty-seven percent attended no more than 5 years of school. Approximately 46% were married before 18 years of age. Approximately half of the interviewed women reported their husbands to be controlling, while 5% noted that their husbands often consumed alcohol (Table 1).

**Table 1:** Sample Characteristics, Overall and According to Forms of Intimate Partner Violence, for Women Interviewed in the National Family Health Survey 4 and 5 (*n* = 122,696), India, 2013–2021

		**Intimate Partner Violence in Previous 12 Months**								
		**Physical**			**Sexual**			**Emotional**		
**Key Exposure Variable**	**Total Sample (*n* = 122,696)** ^**a**^	**Yes (*n* = 26,232)** ^**a**^	**No (*n* = 96,464)** ^**a**^	***P* Value** ^**b**^	**Yes (*n* = 6,099)** ^**a**^	**No (*n* = 116,597)** ^**a**^	***P* Value** ^**b**^	**Yes (*n* = 12,792)** ^**a**^	**No (*n* = 109,904)** ^**a**^	***P* Value** ^**b**^
Precipitation-based drought				<0.001			<0.001			<0.001
No drought	78.46	74.69	79.55		74.84	78.66		76.04	78.77	
Drought	21.54	25.31	20.45		25.16	21.34		23.96	21.23	
Covariates										
Wealth status				<0.001			<0.001			<0.001
Richest	19.97	10.51	22.71		9.55	20.55		11.56	21.05	
Rich	20.95	17.32	22.01		15.09	21.28		17.61	21.38	
Medium	20.82	21.59	20.60		19.60	20.89		22.10	20.66	
Poor	20.12	25.02	18.70		24.58	19.87		23.78	19.65	
Poorest	18.13	25.57	15.98		31.17	17.41		24.95	17.26	
Caste (social status)				<0.001			<0.001			<0.001
Other caste	23.15	15.82	25.31		17.09	23.48		17.12	23.93	
Other backward class^c^	45.96	47.74	45.44		45.38	46.00		45.71	46.00	
Scheduled caste/scheduled tribe	30.89	36.44	29.25		37.53	30.52		37.17	30.07	
Religion				<0.001			0.135			0.017
Hindu	80.08	82.70	79.32		80.39	80.06		81.53	79.89	
Muslim	14.97	13.56	15.38		15.61	14.93		14.69	15.01	
Other	4.95	3.73	5.30		3.99	5.00		3.78	5.10	
Education/years of schooling, years				<0.001			<0.001			<0.001
<5	36.77	48.28	33.45		49.28	36.08		46.79	35.48	
5–12	52.15	46.72	53.72		46.35	52.47		47.95	52.69	
>12	11.08	5.00	12.84		4.36	11.45		5.26	11.83	
Child marriage				<0.001			<0.001			<0.001
Yes	45.65	54.19	43.18		53.65	45.21		52.27	44.80	
No	54.35	45.81	56.82		46.35	54.79		47.73	55.20	
Husband’s occupation				<0.001			<0.001			<0.001
None	2.10	2.05	2.11		2.52	2.07		2.47	2.05	
Agriculture	32.18	38.11	30.47		39.06	31.80		37.24	31.53	
Other	65.72	59.84	67.42		58.42	66.12		60.28	66.42	
Type of residence				<0.001			<0.001			<0.001
Rural	67.63	73.69	65.88		75.75	67.18		72.50	67.01	
Urban	32.37	26.31	34.12		24.25	32.82		27.50	32.99	
Drought-prone area				0.371			0.349			0.028
Yes	30.15	31.99	29.61		29.12	30.20		31.92	29.92	
No	69.85	68.01	70.39		70.88	69.80		68.08	70.08	

^a^ Weighted. %/mean.

^b^ *P* values based on χ^2^ tests.

^c^ “Other backward class” is a collective term used by the Government of India as part of its classifications for population groups.

Approximately 18% of the eligible women from NFHS-4 (2015–2016) were living in areas classified as socioeconomic drought areas by the government of India.

χ^2^ tests showed significant associations of each individual exposure variable and covariate except religion and living in drought-prone areas, for physical IPV and sexual IPV. Emotional IPV was associated with all covariates.

We found significant and positive associations between drought and physical and emotional IPV, for both definitions of drought (Table 2). Associations for sexual IPV were observed only for socioeconomic drought. Women residing in in areas that were classified as having socioeconomic drought were more likely to experience physical IPV (adjusted odds ratio (aOR) =1.34; 95% confidence interval (CI): 1.21, 1.48), sexual IPV (aOR = 1.49; 95% CI: 1.27, 1.74), and emotional IPV (aOR = 1.41; 95% CI: 1.22, 1.63). Similarly, living in areas that experienced precipitation-based drought in the previous year increased the risk of physical IPV (aOR = 1.05, 95% CI: 1.001, 1.10) and emotional IPV (aOR = 1.10; 95% CI: 1.02, 1.16) for married women. The effect sizes of associations were marginally greater for socioeconomic drought, relative to those for precipitation-based drought.

**Table 2:** Logistic Regression Models to Test the Relationship Between Droughts and Forms of Intimate Partner Violence in the Previous 12 Months, India, 2013–2021

	**Intimate Partner Violence in Previous 12 Months by Husband**											
	**Physical**				**Sexual**				**Emotional**			
	**Model 1** ^**a**^		**Model 2** ^**b**^		**Model 1** ^**a**^		**Model 2** ^**b**^		**Model 1** ^**a**^		**Model 2** ^**b**^	
**Exposure Variable**	**OR**	**95% CI**	**aOR**	**95% CI**	**OR**	**95% CI**	**aOR**	**95% CI**	**OR**	**95% CI**	**aOR**	**95% CI**
*Exposure Variable: Socioeconomic Drought*												
No drought	1.00	Referent	1.00	Referent	1.00	Referent	1.00	Referent	1.00	Referent	1.00	Referent
Drought	1.51	1.37, 1.67	1.34	1.21, 1.48	1.79	1.55, 2.07	1.49	1.27, 1.74	1.49	1.30, 1.70	1.41	1.22, 1.63
*Exposure Variable: Precipitation-Based Drought*												
No drought	1.00	Referent	1.00	Referent	1.00	Referent	1.00	Referent	1.00	Referent	1.00	Referent
Drought	1.05	1.01, 1.10	1.05	1.001, 1.10	1.10	1.01, 1.19	1.07	0.97, 1.16	1.10	1.02, 1.15	1.10	1.02, 1.16

Abbreviations: aOR, adjusted odds ratio; CI, confidence interval; OR, odds ratio.

^a^ Unadjusted bivariate.

^b^ Regression models adjusted for wealth, caste, religion, education, husband’s occupation, child marriage, rural/urban residence, drought-prone status, and district-level fixed effects. Models with precipitation-based drought included fixed effects for district level and additionally adjusted for round of data collection.

Stratified analysis showed differences in the relationship between socioeconomic drought and IPV, across years of education, type of residence (rural/urban), and living in drought-prone areas (Table 3). For women residing in non–drought-prone areas (as categorized by the Government of India), an episode of drought was likely to increase the odds of experiencing all 3 forms of violence by around 1.3–1.5 times. These associations were not valid for women residing in drought-prone areas. We did not observe any difference in associations across husband’s occupation or wealth status or for precipitation-based drought (results not shown).

**Table 3:** Adjusted Odds Ratios for Limited Sample Stratified Regression Models to Examine the Relationship Between Socioeconomic Drought and Intimate Partner Violence in the Previous 12 Months, Across Different Social Groups, India, 2013–2021

	**Education** ^**a**^				**Type of Residence** ^**a**^				**Drought-Prone Status** ^**a**^			
	**<5 Years**		**≥5 Years**		**Rural**		**Urban**		**Not Drought-Prone**		**Drought-Prone**	
**Outcome Variable**	**aOR**	**95% CI**	**aOR**	**95% CI**	**aOR**	**95% CI**	**aOR**	**95% CI**	**aOR**	**95% CI**	**aOR**	**95% CI**
Physical IPV	1.42	1.23, 1.63	1.25	1.08, 1.47	1.39	1.21, 1.60	1.34	1.08, 1.66	1.50	1.29, 1.75	1.03	0.79, 1.35
Sexual IPV	1.56	1.26, 1.94	1.32	1.04, 1.69	1.48	1.21, 1.81	1.59	1.15, 2.22	1.59	1.28, 1.98	1.31	0.89, 1.91
Emotional IPV	1.44	1.21, 1.71	1.11	0.90, 1.35	1.31	1.11, 1.55	1.15	0.87, 1.53	1.33	1.12, 1.57	1.21	0.86, 1.71

Abbreviations: aOR, adjusted odds ratio; CI, confidence interval.

^a^ Regression models adjusted for wealth, caste, religion, education, husband’s occupation, child marriage, rural/urban residence, drought-prone status, and district-level fixed effects.

## DISCUSSION

There is a growing interest in and recognition of gender impacts of climate change globally, including violence against women. Overall, and consistent with prior literature, we find that women residing in drought-affected areas are more likely to experience physical, sexual, and emotional partner violence, than those living in non–drought areas. Our findings also confirm the intersectional nature of drought consequences; women who are socially marginalized are at higher risk of IPV victimization due to droughts. Our study emphasizes the need for inclusion of gender perspectives in drought-management strategies, as well as further research to elucidate the mechanisms linking drought and IPV, so we can support better IPV-prevention efforts in the context of growing climate concerns.

Our study highlights the complex nature of droughts. We use 2 definitions of drought. While results from both definitions are similar, we find marginally stronger effect sizes for drought status that is based on socioeconomic consequences of drought (as identified by the government), when compared with a definition of drought that relies only on precipitation patterns. This could be because the risks associated with droughts are a combination of the region’s exposure to the event and the vulnerability of communities to this climate shock, which cannot be captured by rainfall levels alone (12). While decreasing levels of rainfall are concerning, droughts that have an apparent and immediate economic impact in communities may increase women’s vulnerability to experiencing different forms of IPV. However, our data set did not have information on changes in household income or consumption in the previous year, to test this pathway.

The relationship between droughts and IPV differs based on prior history of droughts in a community. We find significant associations between drought and IPV in non–drought-prone areas, and not in drought-prone areas. This might be because severe and frequent drought conditions usually lead to migration of the male members of the communities to nearby places in search of work (27). Additionally, individuals living in areas that have a history of drought are likely better equipped to cope with droughts. Coping strategies can include changing agricultural practices, livelihood diversification, adopting irrigation, and relying on aid (28, 29). In India, support and rehabilitation interventions have been implemented in the areas identified as drought-prone by the government, which can contribute to alleviating some of the drought-induced stress, in addition to providing direct economic relief (30). While our study is not designed to assess these causal relationships between drought-prone status and IPV, future research should consider testing these complex associations.

While our study is timely and significant, it has a few limitations. First, the survey data used in this study relies on self-reported responses and thus is subject to both recall bias and social desirability bias, as well as to the limited generalizability of study findings to India. Second, the survey did not include detailed information about the husband’s occupation and amount of land ownership, to critically examine the role of agriculture in the hypothesized relationships. Our variable of “husband’s occupation” did not allow us to distinguish small- or medium-scale farmers from agricultural landowners. Next, our variable for socioeconomic drought is estimated at the district level, and not at the individual or PSU level. However, drought status is usually consistent within the districts (31), and we have adjusted for spatial clustering of observations within the districts for our regression models. Fourth, the design of the present study includes the use of cross-sectional data, constraining any inferences regarding causality. Relatedly, our study does not capture the long-term associations of droughts. Future research should consider building longitudinal data sets to study this issue, as well as qualitative data to provide insight into how drought affects women and may be creating contexts allowing male violence against partners.

As droughts are expected to increase both in frequency and intensity in India, and IPV can have lasting impacts on a woman’s health, studying the relationship between these phenomena is important for effective policy. Study results emphasize the increased risk of IPV for women living in drought-affected regions in India; effect sizes for the associations are stronger in cases where resources are inadequate for individuals to cope with such climate shocks. The findings also highlight the need for adoption of gender perspectives in strategies for disaster management and preparedness broadly. It is important for IPV-prevention interventions to take into account climate events when identifying their target geographies, in a context of increasing drought due to climate change.

## Supplementary Material

Web_Material_kwad222
