# Supplementary material for: Climate and Gender: Association Between Droughts and Intimate Partner Violence in India
Source: Am J Epidemiol. 2023 Nov 15;193(4):636–45. doi: 10.1093/aje/kwad222 (PMC10999644; doi:10.1093/aje/kwad222)
Supplement: Web_Material_kwad222 [file web_material_kwad222.zip › kwad222 Dehingia Web Material Final.pdf]

Web Material

Climate and gender: association between droughts and intimate partner violence in India

Nabamallika Dehingia, Lotus McDougal, Jay G. Silverman, Elizabeth Reed, Lianne Urada, Julian McAuley, Abhishek Singh, and Anita Raj

Table of Contents

Web Figure 1 ……………………………………………………………………2

Web Figure 2 ……………………………………………………………………3

Web Table 1 ……………………………………………………………………4

Web Figure 1. Annual precipitation levels in India from 1990-2020

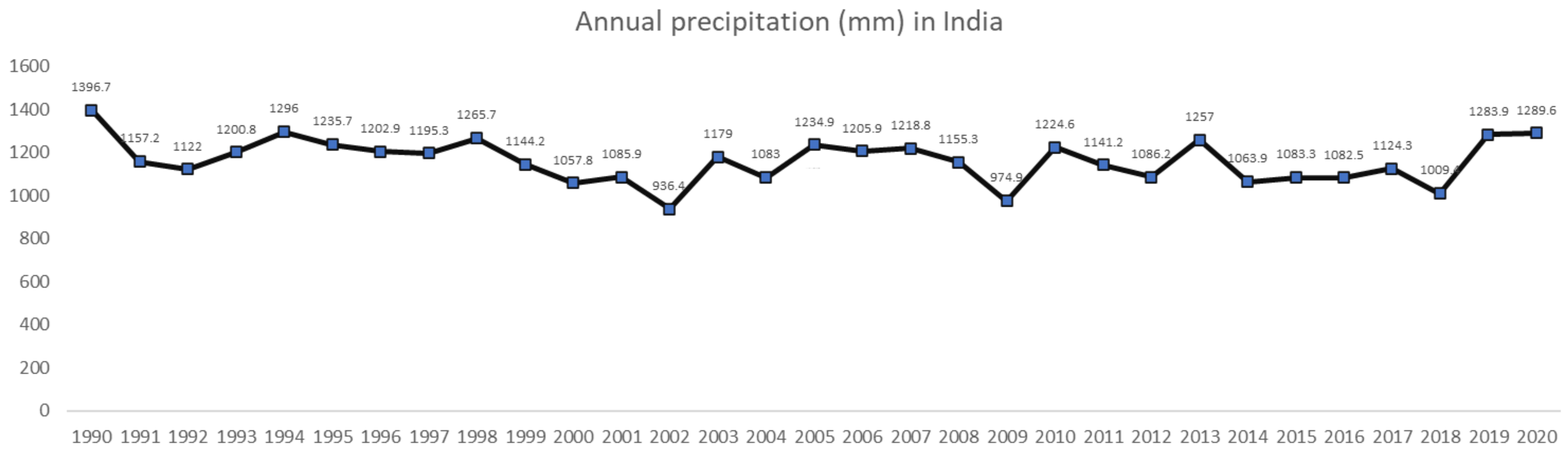

Web Figure 2. Direct Acyclic Graph (DAG) for the models examining the relationship between drought and IPV. DAG made using *daggity.net*

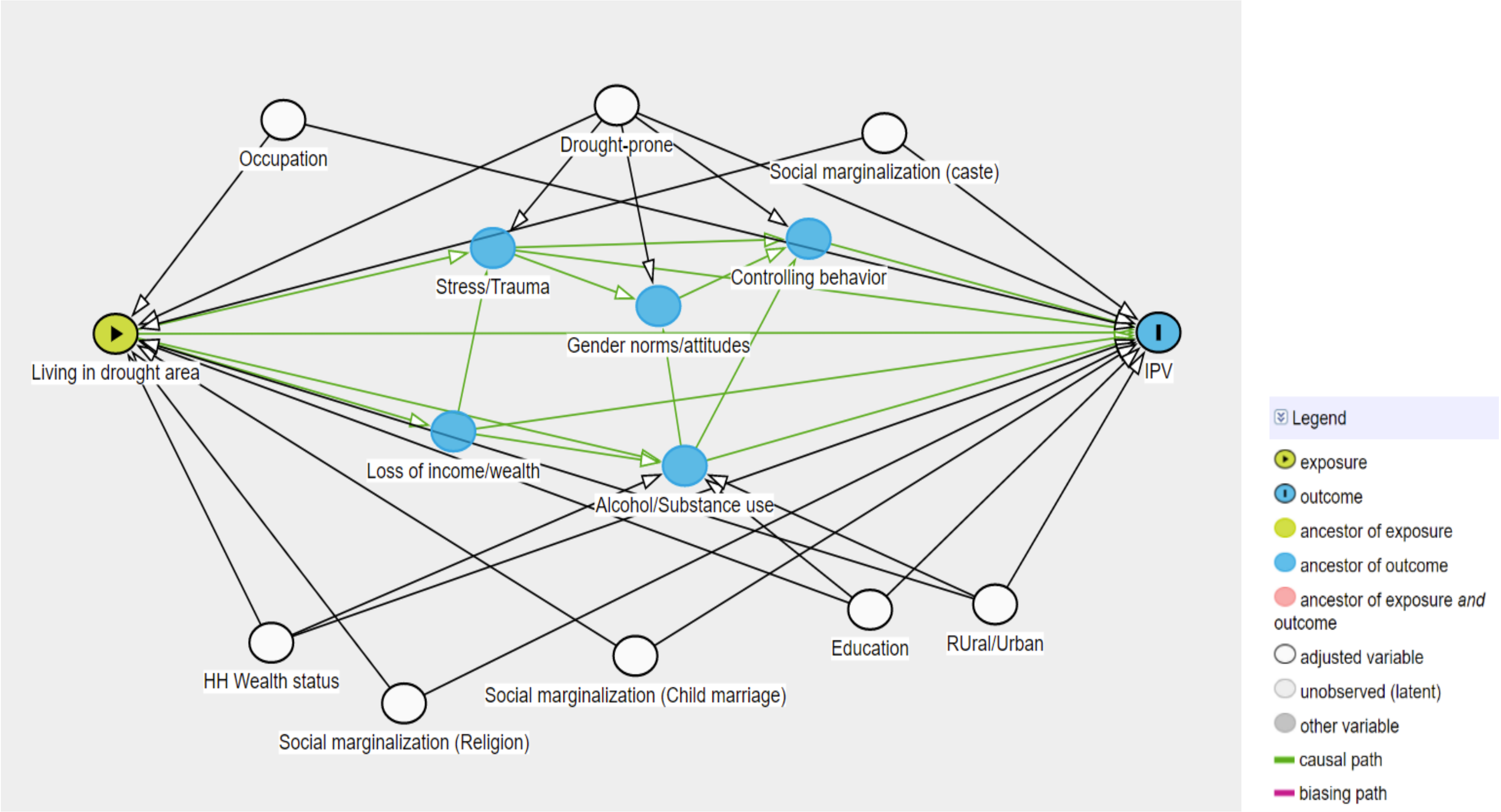

Web Table 1: Logistic regression models to test the relationship between precipitation-based droughts and intimate partner violence in the past 12 months, with different time lag between drought and IPV

	Model: Two-year lag between drought and IPV<br><br>Adjusted Odds Ratio (aOR)		Model: No lag between drought and IPV (concurrent year)<br><br>Adjusted Odds Ratio (aOR)
**Outcome: Physical violence**			
No drought	REF		REF
Drought	1.11 (1.02- 1.21) **		1.02 (0.96-1.09)
**Outcome: Sexual violence**			
No drought	REF		REF
Drought	1.11 (0.97- 1.27)		1.09 (0.97- 1.21)
**Outcome: Emotional violence**			
No drought	REF		REF
Drought	1.17 (1.04- 1.30) ***		0.97 (0.89- 1.05)

* p<0.10; **p<0.01; ***p<0.001
